# Countdown to 2030: eliminating hepatitis B disease, China

**DOI:** 10.2471/BLT.18.219469

**Published:** 2019-01-28

**Authors:** Jue Liu, Wannian Liang, Wenzhan Jing, Min Liu

**Affiliations:** a Department of Epidemiology and Biostatistics, School of Public Health, Peking University, No.38, Xueyuan Road, Haidian District, Beijing 100191, China.; b Department of Healthcare Reform, National Health Commission of the People’s Republic of China, Beijing, China.

## Abstract

Hepatitis B virus (HBV) infection is a major public health problem worldwide. China has the world’s largest burden of HBV infection and will be a major contributor towards the global elimination of hepatitis B disease by 2030. The country has made good progress in reducing incidence of HBV infection in the past three decades. The achievements are mainly due to high vaccination coverages among children and high coverage of timely birth-dose vaccine for prevention of mother-to-child transmission of HBV (both > 95%). However, China still faces challenges in achieving its target of 65% reduction in mortality from hepatitis B by 2030. Based on targets of the World Health Organization’s *Global health sector strategy on viral hepatitis 2016–2021*, we highlight further priorities for action towards HBV elimination in China. To achieve the impact target of reduced mortality we suggest that the service coverage targets of diagnosis and treatment should be prioritized. First, improvements are needed in the diagnostic and treatment abilities of medical institutions and health workers. Second, the government needs to reduce the financial burden of health care on patients. Third, better coordination is needed across existing national programmes and resources to establish an integrated prevention and control system that covers prevention, screening, diagnosis and treatment of HBV infection across the life cycle. In this way, progress can be made towards achieving the target of eliminating hepatitis B in China by 2030.

## Résumé

Les infections par le virus de l'hépatite B (VHB) constituent un problème de santé publique majeur à l'échelle mondiale. La Chine est le pays le plus lourdement touché par les infections par le VHB et sa contribution sera donc essentielle pour atteindre l'objectif d'élimination de l'hépatite B dans le monde à l'horizon 2030. Au cours des trente dernières années, le pays a fait des progrès notables en matière de réduction de l'incidence des infections par le VHB. Ces avancées sont principalement dues aux forts niveaux de couverture vaccinale des enfants et d'administration de la dose vaccinale à la naissance visant à prévenir la transmission du VHB de la mère à l'enfant (>95% dans les deux cas). Néanmoins, la Chine doit encore relever des défis de taille pour atteindre l'objectif de réduction de 65% de la mortalité due à l'hépatite B d'ici à 2030. À partir des cibles définies dans la *Stratégie mondiale du secteur de la santé contre l’hépatite virale, 2016-2021* de l'Organisation mondiale de la Santé, nous avons identifié les actions à mener prioritairement en vue de l'élimination du VHB en Chine. Pour atteindre l'objectif de réduction de la mortalité, nous suggérons d'orienter en priorité les efforts sur l'amplification de la couverture des services de diagnostic et de traitement. Premièrement, des améliorations sont requises au niveau des capacités diagnostiques et de traitement des institutions médicales et des agents de santé. Deuxièmement, le gouvernement doit réduire la charge financière des soins de santé qui pèse sur les patients. Troisièmement, une meilleure coordination est nécessaire entre les ressources et les programmes nationaux existants, en vue d'établir un système intégré de prévention et de lutte englobant la prévention, le dépistage, le diagnostic et le traitement des infections par le VHB à tous les âges de la vie. De nouveaux progrès pourraient ainsi être faits en vue d'atteindre l'objectif d'élimination de l'hépatite B en Chine à l'horizon 2030.

## Resumen

La infección por el virus de la hepatitis B (VHB) es un gran problema de sanidad pública en todo el mundo. China tiene la mayor carga de infección por VHB en el mundo y será uno de los principales contribuyentes a la eliminación mundial de la enfermedad de la hepatitis B para 2030. En las últimas tres décadas, el país ha hecho grandes progresos en la reducción de la incidencia de la infección por el VHB. Los logros se deben principalmente a la alta cobertura de vacunación entre los niños y a la alta cobertura de la vacuna de dosis oportuna al nacer para la prevención de la transmisión maternoinfantil del VHB (ambas > 95 %). Sin embargo, China sigue teniendo dificultades para alcanzar su objetivo de reducir en un 65 % la mortalidad por hepatitis B para 2030. Basados en los objetivos de la Organización Mundial de la Salud sobre la *Estrategia global del sector sanitario para la hepatitis viral 2016-2021*, destacamos otras prioridades de acción para la eliminación del VHB en China. Para lograr el objetivo de impacto de la reducción de la mortalidad, sugerimos que se prioricen los objetivos de cobertura de los servicios de diagnóstico y tratamiento. En primer lugar, es necesario mejorar la capacidad de diagnóstico y tratamiento de las instituciones médicas y los trabajadores sanitarios. En segundo lugar, el gobierno debe reducir la carga financiera de la atención sanitaria para los pacientes. En tercer lugar, se necesita una mejor coordinación entre los programas y recursos nacionales existentes para establecer un sistema integrado de prevención y control que abarque la prevención, el cribado, el diagnóstico y el tratamiento de la infección por VHB a lo largo de todo el ciclo de vida. De esta manera, se puede avanzar hacia el objetivo de eliminar la hepatitis B en China para 2030.

## ملخص

تمثل عدوى فيروس الالتهاب الكبدي ب (HBV) إحدى المشكلات الصحية العامة الكبيرة حول العالم. تنوء الصين بأكبر حصة عالمية من عدوى فيروس الالتهاب الكبدي، وستكون مساهماً رئيسياً في القضاء على مرض الالتهاب الكبدي ب حول العالم بحلول عام 2030. لقد حققت الدولة تقدماً جيداً في الحد من انتشار عدوى فيروس الالتهاب الكبدي ب في العقود الثلاثة الماضية. وتعود تلك الإنجازات بشكل رئيسي إلى ارتفاع التغطية بالتطعيم بين الأطفال، والتغطية العالية لتطعيم جرعة الولادة في وقتها المناسب، للوقاية من انتقال فيروس الالتهاب الكبدي ب من الأم إلى الطفل (كلاهما > 95٪). ومع ذلك، لا تزال الصين تواجه تحديات في تحقيق هدفها بتخفيض نسبة الوفيات الناجمة عن الالتهاب الكبدي ب بنسبة 65٪ بحلول عام 2030. واستناداً إلى أهداف استراتيجية قطاع الصحة العامة لمنظمة الصحة العالمية بشأن الالتهاب الكبدي الفيروسي للفترة من 2016 إلى 2021، فإننا نسلط الضوء على أولويات إضافية للعمل على التخلص من فيروس الالتهاب الكبدي ب في الصين. ولتحقيق هدف التأثير المتمثل في خفض معدل الوفيات، نقترح منح أولوية أهداف تغطية الخدمة لكل من التشخيص والعلاج. أولاً، هناك حاجة إلى التحسينات في قدرات التشخيص والعلاج للمؤسسات الطبية والعاملين الصحيين. ثانيا، تحتاج الحكومة إلى الحد من العبء المالي للرعاية الصحية على المرضى. ثالثاً، هناك حاجة إلى تنسيق أفضل عبر البرامج والموارد الوطنية الحالية لإنشاء نظام متكامل للوقاية والتحكم يغطي الوقاية من فيروس الالتهاب الكبدي ب وفحصه وتشخيصه وعلاجه طوال دورة الحياة. وبهذه الطريقة، يمكن إحراز تقدم نحو تحقيق هدف القضاء على الالتهاب الكبدي ب في الصين بحلول عام 2030.

## 摘要

乙型肝炎病毒 (HBV) 感染是一个严重的全球公共卫生问题。中国是世界上乙型肝炎病毒 (HBV) 感染负担最重的国家，也是实现 2030 年前全球消除乙肝目标的主要贡献者。近 30 年来，我国的乙肝病毒感染发生率呈现显著下降趋势。取得这些成就的主要原因在于儿童疫苗接种的覆盖率高，以及新生儿在出生时及时接种疫苗【预防乙型肝炎病毒 (HBV) 母婴传播】的覆盖率高（均高于 95%）。然而，中国要在 2030 年前实现乙肝死亡率降低 65% 这一目标，仍面临巨大挑战。根据世界卫生组织《2016-2021 年全球卫生部门病毒性肝炎战略》目标，我们强调在中国进一步消除乙型肝炎病毒 (HBV) 的重点行动。为了实现降低死亡率的影响指标，我们建议优先考虑诊断与治疗的服务覆盖率目标。首先，提高医疗机构和医疗工作者的诊断与治疗能力。其次，政府需要减轻患者接受治疗的经济负担。第三，更好地协调现有国家规划与资源之间的关系，以建立涵盖整个生命周期的乙型肝炎病毒 (HBV) 感染的预防、筛查、诊断和治疗的综合预防和控制系统。这样，中国才能在 2030 年消除乙肝的目标上取得进步。

## Резюме

Инфекция вирусом гепатита В (HBV) представляет собой одну из самых серьезных проблем общественного здравоохранения во всем мире. Бремя этого заболевания в Китае крупнейшее в мире, и эта страна должна будет внести основной вклад в ликвидацию гепатита В в масштабах планеты к 2030 году. Китай хорошо продвинулся на пути снижения уровня инфицирования HBV за последние три десятилетия. Этими достижениями он обязан в основном высокому покрытию вакцинированием среди детей и высокому уровню своевременной вакцинации в момент рождения для предотвращения передачи гепатита В от матери ребенку (оба показателя превышают 95%). Однако Китай все еще сталкивается с затруднениями в достижении своей цели — снижения смертности от гепатита В на 65% к 2030 году. Основываясь на целях *Глобальной стратегии сектора здравоохранения по вирусному гепатиту 2016–2021 гг.* Всемирной организации здравоохранения, авторы обозначают будущие приоритеты для действий в направлении ликвидации HBV в Китае. Для того чтобы достичь заданной цели снижения смертности, авторы предлагают выдвинуть на первое место цели, связанные с охватом диагностикой и лечением. Прежде всего нужно достичь улучшений в части возможностей для диагностики и лечения как для медицинских учреждений, так и для медицинских работников. Во-вторых, правительству следует снизить бремя финансовых расходов по здравоохранению, которое возлагается на пациентов. В-третьих, существующие национальные программы и ресурсы должны лучше координироваться для создания единой системы профилактики и контроля, которая бы охватывала задачи профилактики, скринингового обследования, диагностики и лечения инфекции HBV на протяжении жизненного цикла. Таким путем можно достичь прогресса в достижении цели ликвидации гепатита В в Китае к 2030 году.

## Introduction

**Fig. 1:**
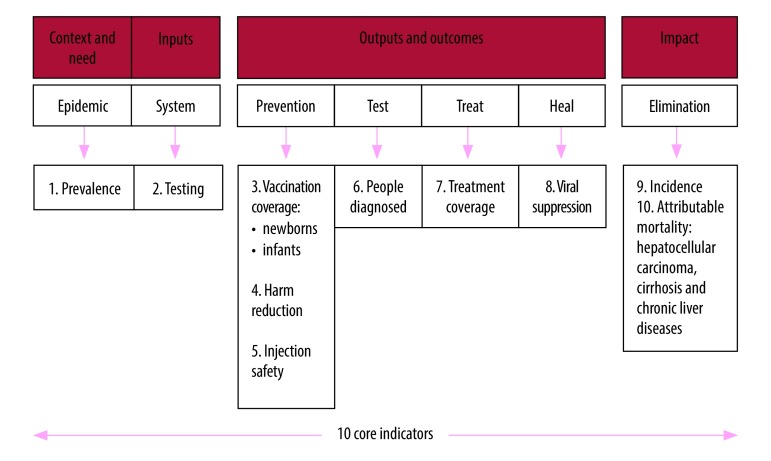
WHO evaluation framework for hepatitis B virus elimination

Hepatitis B virus (HBV) infection is a major public health problem worldwide. The World Health Organization (WHO) estimated that 257 million people were living with chronic HBV infection in 2015 and that hepatitis B results in 887 000 deaths every year worldwide.^1^^,^^2^ To take action on sustainable development goal 3.3 on combating hepatitis, the World Health Assembly approved the global health sector strategy to eliminate viral hepatitis as a public health threat by 2030, with a target of reducing new infections by 90% and mortality by 65%.^3^ According to the global strategy, elimination of hepatitis B disease requires synergy across five core interventions: (i) immunization against hepatitis B; (ii) prevention of mother-to-child transmission (PMTCT) of HBV; (iii) blood and injection safety; (iv) harm reduction services for people who inject drugs; and (v) increased testing and treatment.^3^ To evaluate the global strategy, WHO also proposed a monitoring and evaluation framework for hepatitis B with 10 core indicators (Fig. 1).^4^

WHO: World Health Organization.

Source: *Monitoring and evaluation for viral hepatitis B and C: recommended indicators and framework*.^4^

China has the world’s largest burden of HBV infection and will be a major contributor to the global elimination of hepatitis B by 2030.^5^^,^^6^ The country has made good progress in combating hepatitis B in the past three decades. However, with the largest population in the world (1.39 billion in 2017), the absolute number of HBV-infected people in China is large at around 70 million.^7^ China still faces challenges to achieving the goal of hepatitis B elimination by 2030.^4^ In this paper we summarize China’s achievements and gaps in progress towards elimination of hepatitis B by 2030. We highlight further priorities for action and make suggestions on the implementation roadmap for hepatitis B elimination in China.

## Approach

We conducted an online search for articles published before 20 November 2018. We searched the PubMed®, EMBASE® and the Cochrane Library databases for English language articles and the China National Knowledge Infrastructure and Wanfang databases for Chinese language articles. We used the search terms “hepatitis B”, “elimination of hepatitis”, “epidemiology”, “vaccination”, “PMTCT”, “blood safety”, “safe injection”, “harm reduction”, “diagnosis” and “treatment.” We also reviewed reports and health statistical yearbooks from WHO, the Joint United Nations Programme on HIV/AIDS and the Chinese government published over the past three decades. We based our analysis on the core indicators of the framework of the WHO *Monitoring and evaluation for viral hepatitis B and C evaluation*^4^ and the targets of the *Global health sector strategy on viral hepatitis 2016‒2021*.^3^

## Progress

### Prevalence

In the past three decades, China has changed from a highly endemic to an intermediate endemic area for HBV infection.^7^^,^^8^ According to the data from national surveys in China, the weighted prevalence of hepatitis B surface antigen (HBsAg) adjusted for people aged 1–59 years declined from 9.8% in 1992 to 7.2% in 2006.^9^ Weighted HBsAg prevalence among people aged 1–29 years declined during 1992–2006, from 10.1% to 5.5% and during 2006–2014, from 5.5% to 2.6%.^8^ At present, it is estimated that there are about 70 million HBsAg carriers (5–6% prevalence).^7^^,^^10^

### Testing and diagnosis

Testing and diagnosis of HBV infection is the gateway for patients to access both prevention and treatment services, and is a crucial component of an effective response to the hepatitis B epidemic.^11^ In 2017, China updated its national viral hepatitis prevention and control plan (2017–2020).^12^ To increase the coverage of testing, the Chinese government requires medical institutions to screen for hepatitis B in all pregnant women during antenatal care and in patients who are undergoing surgery, hospitalization, haemodialysis or invasive diagnosis and treatment.^12^ The coverage of diagnosis has been improved due to the increasing proportion of pregnant women attending antenatal care (from 69.7% of 11.75 million live births in 1992 to 96.6% of 18.47 million live births in 2016) and the number of surgeries among inpatients (from 14.0 million in 2002 to 50.8 million in 2016).^13^ In addition, China launched the national preconception health examination project in 2010 to provide free health check-ups for reproductive couples (including free HBV serological testing) in 100 counties and then expanded it nationally in 2013.^14^ A study in rural China reported that nearly 1 in 10 couples preparing for pregnancy (202 816 out of 1 936 801 couples) are either discordant or both positive for HBsAg.^15^ From 2010 to 2016, the Chinese government allocated 7.25 billion Chines yuan (¥; ¥ 1equivalent to 0.15 United States dollars) for the national preconception health examination project and screened around 60.5 million couples.^16^^,^^17^

### Vaccination coverage

**Fig. 2:**
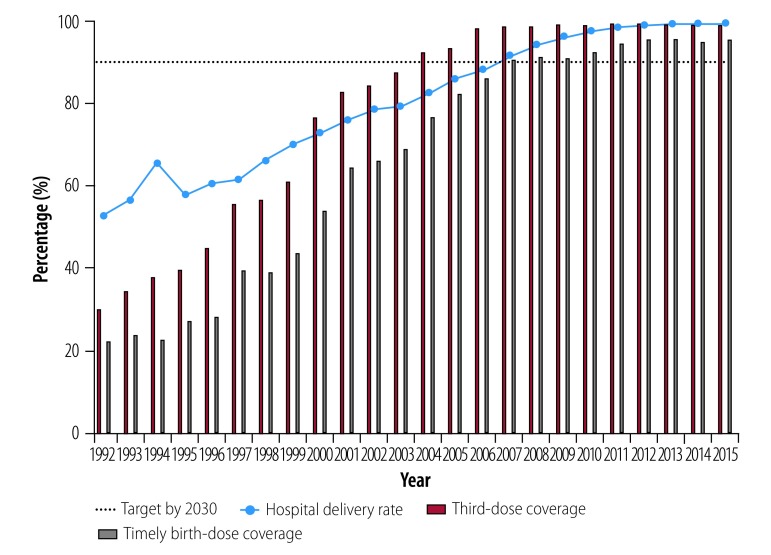
Target and actual hepatitis B virus vaccination coverage and hospital delivery rate in China, 1992–2015

To combat hepatitis B, the Chinese government has made timely vaccination on newborns and infants its highest priority.^9^ In 1992, China was among the first developing countries to enact a universal hepatitis B vaccination programme for newborns and infants.^9^ China has integrated hepatitis B vaccine into the national expanded programme on immunization and provided free vaccination since 2002.^9^ The Chinese government allocated approximately ¥ 5.3 billion for neonatal hepatitis B vaccination from 1992 to 2005 and ¥ 15 billion for the procurement of national immunization vaccines and syringes.^18^^,^^19^ From 2009 to 2011, a catch-up campaign was launched for children younger than 15 years, which succeeded in vaccinating nearly 68 million children.^20^ With the support of national financial funds, vaccination coverage has been effectively guaranteed. Reported coverage of three doses of hepatitis B for infants has increased from 30.0% in 1992 to 99.6% in 2015, and timely birth-dose coverage has increased from 22.2% in 1992 to 95.6% in 2015 (Fig. 2).^20^^–^^24^ Meanwhile, timely birth-dose coverage is also guaranteed by the high hospital delivery rate which has increased from 52.7% of live births in 1992 to 99.8% of live births in 2016.^13^ Hepatitis B three-dose coverage and timely birth-dose coverage in China have both achieved the service coverage target of 90% in the elimination of hepatitis B.

Data sources: Chinese Centre for Disease Control and Prevention^20^^–^^24^ and *China health statistics yearbook*.^13^

Notes: Third-dose coverage is the number of infants receiving the third dose of HBV vaccine as a percentage of the total number of live births. Timely birth-dose coverage is the number of infants receiving the birth dose of HBV vaccine within 24 hours as a percentage of the total number of live births. Target by 2030 is both third-dose coverage and timely birth-dose coverage of 90% by 2030. Hospital delivery rate is the number of live births in institutions with midwifery-qualified staff as a percentage of the number of all live births.

Mother-to-child transmission is the main route of HBV transmission in high-endemic areas and is responsible for an estimated 30–50% new infections in China.^25^ In 2011, China conducted the national programme for integrated PMTCT of HIV, syphilis and hepatitis B to provide free hepatitis B immunoglobulin within 12 hours after birth and three doses of vaccine for children born to HBV-infected women.^26^ The government allocated about ¥ 3.4 billion and covered 1156 out of 2853 counties (41%) in China from 2011 to 2013.^27^ The coverage of hepatitis B immunoglobulin in the national integrated PMTCT programme reached 774 916 people (97.7%) in 2013.^28^ Since 2015, the national integrated PMTCT programme has expanded nationwide, and the Chinese government continues to invest ¥ 1.4 billion each year to provide free screening for mothers and comprehensive intervention services (including free hepatitis B immunoglobulin) to maintain the progress.^29^ The government has achieved the service coverage targets for hepatitis B vaccination and PMTCT of HBV (both are above 90% coverage; Table 1).

**Table 1:** Gaps and priorities in progress towards elimination of hepatitis B in China by 2030

Target area^a^	Baseline values			Targets				Assessment of gaps and priorities for China		
	Global, 2015^3^	China (year)		WHO, 2020	WHO, 2030	China, 2020^12^		Gaps to 2030^b^	Efforts required	Priority^c^
**Impact targets**										
Incidence: New cases of chronic viral hepatitis B infections^d^	1.3%^3^	0.3%^8^ (in 2014)		30% reduction (equivalent to 1% prevalence of HBsAg among children)	90% reduction (equivalent to 0.1% prevalence of HBsAg among children)	Maintain < 1%		Small	More	NA
Mortality: Viral hepatitis B deaths	0.887 million^2^	0.308 million^6^ (in 2016)		10% reduction	65% reduction	NA		Large	Maximum	Highest
**Service coverage targets**										
Hepatitis B virus vaccination: Childhood vaccine coverage (third dose coverage)	82%^3^	99.6%^22^ (in 2015)		90%	90%	Maintain > 95%		None	Maintain	NA
Prevention of hepatitis B virus mother-to-child transmission: Hepatitis B virus birth-dose vaccination coverage or other approach to prevent mother-to-child transmission	38%^3^	95.6%^22^ (in 2015)		50%	90%	Maintain > 90%		None	Maintain	NA
Blood safety: % of donations screened in a quality-assured manner	89%^3^	100%^30^ (in 2015)		95%	100%	Nucleic acid test 100%		None	Maintain	NA
Safe injections: % of injections administered with safety-engineered devices in and out of health facilities	5%^3^	86.5%^31^ (in 2015)^e^		50%	90%	NA		Small	More	NA
Harm reduction: No. of sterile needles and syringes provided per person who injects drugs per year	20^3^	208^31^ (in 2015)		200	300	NA		Small	More	NA
Viral hepatitis B diagnosis: % of chronic hepatitis infections diagnosed	< 5%^3^	19%^10^ (in 2016)		30%	90%	NA		Large	Maximum	Highest
Viral hepatitis B treatment: % of eligible persons with chronic hepatitis B virus infection treated	< 1%^3^	10–11%^5^^,^^10^ (in 2016)		Cumulative target is 5 million people treated for HBV	80%	NA		Large	Maximum	Highest

HBV: hepatitis B virus; HBsAg: hepatitis B surface antigen; NA: not applicable; WHO: World Health Organization.

^a^ Targets of the WHO global health sector strategy.^3^

^b^ Large gap defines the current baseline value differs from the target value by more than 50%. Small gap defines the gap is less than 50%.

^c^ Priority to achieve the goal of elimination hepatitis by 2030 in China.

^d^ Prevalence of HBsAg in children younger than 5 years.

^e^ Percentage of safe injections among people who inject drugs.

### Harm reduction

Safe injections are defined as injections administered with safety-engineered devices within and outside health facilities. In 2000, China passed the specifications on nosocomial infection management and methods for supervision to ban the reuse of disposable sterile medical devices in health facilities.^29^^,^^32^ Meanwhile, to eliminate unsafe vaccine injections, auto-disposable syringes became available for vaccine injections in China since 2007 and all reusable injection equipment was eliminated by 2010.^33^

Sharing injection equipment by people who inject drugs leads to transmission via contaminated injection paraphernalia of blood-borne viruses, including HBV. There are an estimated 2.56 million injection drug users in China, who account for 16.4% of the 15.65 million global number of people who inject drugs.^31^ To date, 23.4% (0.6 million) of Chinese injection drug users are HBsAg-positive.^31^ In 2014, 11 million needle syringes were distributed at 814 needle and syringe programme sites.^34^ In 2015, the annual number of needles and syringes distributed was estimated as 208 per person who injects drugs (versus 300 per person who injects drugs in 2030 targets) and safe injections among people who inject drugs was 86.5% (versus 90% in 2030 targets).^35^

### Antiviral treatment

Antiviral treatment in patients with chronic hepatitis B is typically a lifelong commitment. Available treatment options for chronic hepatitis B include interferon-α, pegylated interferon-alfa and nucleoside analogues. Entecavir, tenofovir or pegylated interferon-α is recommended for treatment-naïve patients.^36^ According to data from the China registry of hepatitis B, by 2016 88.7% of 33 533 patients treated for chronic hepatitis B were receiving nucleoside analogues therapy.^37^ Entecavir, lamivudine, adefovir, telbivudine and tenofovir accounted for 51.2%, 18.8%, 16.1%, 12.5% and 1.4% of chronic hepatitis B patients receiving nucleoside analogues therapy, respectively.^37^ Compliance is influenced by treatment affordability. The government invested more than ¥ 3 trillion into the health system between 2009 and 2014 to expand the coverage of social insurance schemes to reach 1.3 billion people and establish a national essential medicines system.^30^ Tenofovir was added to the national basic medical insurance reimbursement list in 2017, and the average daily cost sharply reduced from ¥ 49.0 to ¥ 16.6 per day.^38^ Until 2017, all of the antiviral drugs recommended by the Chinese guidelines (interferon-alfa, pegylated interferon-alfa and five nucleoside analogues) are included in the national basic medical insurance reimbursement list as partial out-of-pocket expenses, and this has contributed to the improvement of treatment coverage.^36^^,^^38^

### Incidence

According to the WHO global strategy, the target of 90% reduction in incidence by 2030 is equivalent to a 0.1% prevalence of HBsAg among children.^3^ In China, the weighted prevalence of HBsAg has decreased by 97% (from 9.9% in 1992 to 0.3% in 2014) among children younger than 5 years.^8^ This shows that China has achieved the target of 30% reduction in incidence, equivalent to 1% prevalence of HBsAg among children, by 2020.^1^^,^^3^ The success can be attributed to the effective implementation of the universal hepatitis B vaccination programme for infants and the national integrated PMTCT programme among children born to HBV-infected women. 

## Challenges

We have analysed progress towards elimination of hepatitis B by 2030 in China according to the impact and coverage targets of the WHO global strategy. As shown in Table 1, there are small or no gaps in meeting the targets of reducing incidence, vaccination, PMTCT, blood safety, safe injections and harm reduction. However, there are large gaps towards reaching the targets of reducing mortality and increasing coverage of diagnosis and treatment, showing that China still faces challenges in elimination of HBV infection by 2030.

### Reducing mortality

**Fig. 3:**
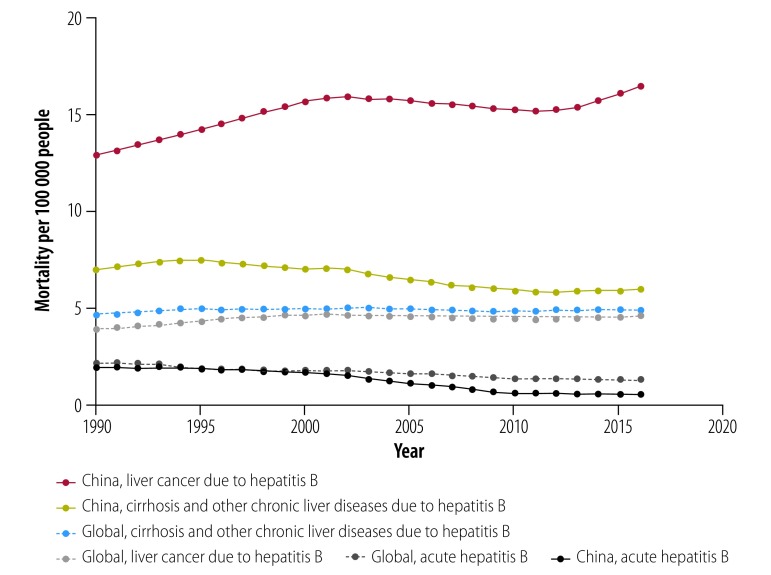
Mortality due to hepatitis B disease in China and globally, 1990–2016

**Fig. 4:**
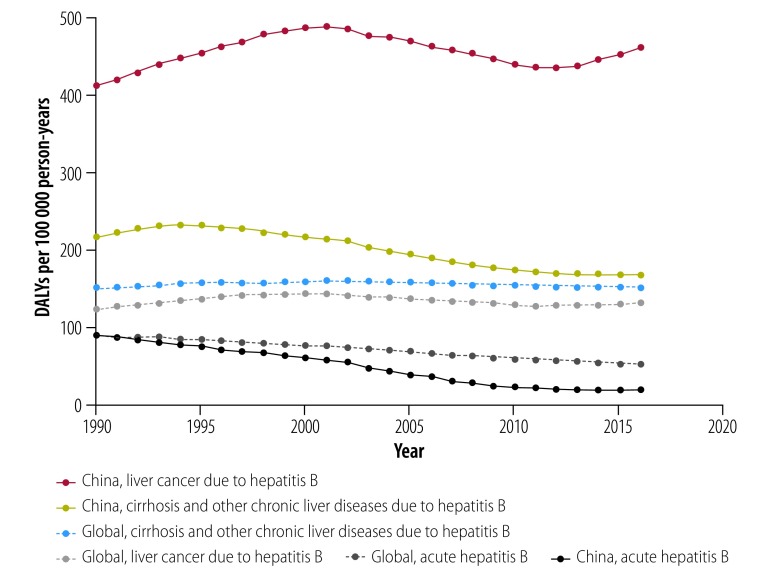
Disability-adjusted life-years attributed to hepatitis B disease in China and globally, 1990–2016

Globally, HBV-related liver disease represents the seventh highest cause of mortality worldwide.^39^ Deaths due to HBV-related liver diseases in China (0.308 million deaths per year) account for more than 30% of the global mortality from HBV (0.887 million deaths per year). At present, estimations suggest that in China there are 20–30 million people with chronic hepatitis B, 1 million with liver cirrhosis and 0.3 million with hepatocellular carcinoma caused by hepatitis B.^40^ The mortality rate of HBV-related cirrhosis and other chronic liver diseases decreased from 7.45 to 5.82 per 100 000 people from 1990 to 2016, and the mortality rate of HBV-related liver cancer in China increased from 12.88 per 100 000 people in 1990 to 16.42 per 100 000 people in 2016 (Fig. 3).^6^ The annual disability-adjusted life-years of liver cancer caused by hepatitis B in China from 1990 to 2016 was consistently higher than the global level: 400 versus 130 per 100 000 person-years (Fig. 4).^6^ Low coverage of diagnosis and treatment of hepatitis B has contributed to the challenges of reducing mortality.

Data source: *Global health data results tool*.^6^

DALYs: disability adjusted life-years.

Data source: *Global health data results tool*.^6^

### Increasing coverage

High coverage of hepatitis B diagnosis and treatment have become the most difficult targets to achieve globally. Currently, it is estimated that only 16.1 million (19%) of chronic HBV-infected people are diagnosed in China (versus 90% in the 2030 targets),^10^ and only 2.8 million (10–11%) of patients with chronic hepatitis B are currently receiving the needed treatment (versus 80% in 2030 targets).^5^^,^^10^ Several reasons can explain low coverage of diagnosis, inadequate accessibility and poor adherence to treatment. First, the distribution of medical resources in China is still not geographically equal, and there are discrepancies across regions in the diagnostic abilities of medical institutions and health workers. Diagnosis coverage is affected by the number of facilities with capacity to test for HBV. A nationwide provider survey conducted in 2015 among 149 community health centres in China showed that the majority of centres (119; 80%) offered HBV testing, and doctors felt that the major barriers for not offering testing were lack of training (444/827; 54%) and financial support (187/827; 23%).^41^ Second, both treatment rates and patient compliance are hindered by the high cost of antiviral treatment and the limited proportion of medical insurance payments. Third, institutional and governance fragmentation hampers concerted efforts made to combat hepatitis B.^30^ Different government agencies are involved in combating hepatitis B, while each pursues its bureaucratic objectives with a limited vision beyond its own sphere of decision-making.^30^ The Chinese government has invested a large amount of money on free-of-charge vaccination and testing services in the expanded programme on immunization, the national preconception health examination project and the national integrated PMTCT programme, however, these programmes, operate in relative isolation and not linked with each other.

## Priorities for action

From the analysis of gaps towards meeting the 2030 targets (Table 1), we suggest that priority should be given to improving the service coverage targets of hepatitis B diagnosis and treatment to achieve the impact target of 65% reduction in mortality.

First, exploring the tiered mode of health-care delivery and improve the diagnostic and treatment abilities of medical institutions and health workers for chronic hepatitis B in China is necessary. In March 2018, the National Health and Family Planning Commission of China was renamed as the National Health Commission and prioritized universal health care. This prioritization will help the nation achieve its Healthy China 2030 plan, including HBV elimination by 2030. For instance, health-care workers could provide continuous and comprehensive care on the diagnosis, treatment, nursing and rehabilitation of chronic hepatitis B patients. This care strategy could be achieved via vertical integration of care provided within hospitals, primary care and communities by establishing multidisciplinary teams, evidence-based integrated clinical pathways and referral systems, and individualized care plans for chronic hepatitis B patients.^30^ By taking advantage of health-care system reform, the Chinese government could reshape the delivery system for HBV-related services and strengthen the professional training of health workers to provide an optimal service guarantee for HBV elimination by 2030.

Second, providing optimal and affordable financial support for the elimination of hepatitis B by 2030 and reducing the financial burden of health care for patients is necessary. The percentage of out of pocket payments made by patients affects accessibility and utilization of health-care services for households.^42^ In November 2018, three first-line HBV therapeutic drugs (entecavir, plustenofovir and pegylated interferon-α) were newly included in the 2018 edition of the China national essential drugs list.^43^ The next step is to add these first-line therapeutic drugs into the medical insurance catalogue or adjust the reimbursement category so that all the costs are reimbursed by the national basic medical insurance. Medical costs for the diagnosis and treatment of liver disease related to hepatitis B could be reduced to improve the affordability of individual therapies. This reduction could be done by taking full advantage of all the basic medical insurance categories in China. In addition, social resources (for example, commercial health insurance) could be encouraged as a good supplement to the basic medical insurance. Patients with better economic conditions could choose commercial health insurance to cover the costs of diagnosis and treatment of HBV infection, liver cirrhosis, hepatocellular carcinoma and other HBV-related diseases, which the basic medical insurance could not cover.

Third, by increasing the service coverage targets of diagnosis and treatment, China could integrate existing national programmes related to hepatitis B; optimize health-care resource allocation; reduce inefficient service delivery and fragmentation; and establish an integrated care model to combat hepatitis B across the life cycle from newborn to adulthood.^30^ These actions could provide the optimal implementation path for achieving the 2030 HBV goal. Specifically, the Chinese government could combine the national integrated programme for PMTCT of HIV, syphilis and hepatitis B with the national free preconception health examination project. This action would establish a comprehensive prevention and control strategy by treating the family as a unit through the continuum of detection, immunization, treatment and elimination of hepatitis B. By integrating existing national programmes covering infancy and the pre-pregnancy and perinatal periods, health-care services in China could provide timely counselling for infected individuals on how to prevent hepatitis disease transmission and progression as well as identify and vaccinate susceptible contacts. Young adults diagnosed with infection in the national free preconception health examination project and who might not have yet progressed to late-stage liver disease could receive more timely and effective treatments in the early stage.^44^

Social drivers can also affect progress on HBV elimination, such as patients’ educational level, occupation and income, and the country’s reimbursement policies. Previous studies showed that knowledge about HBV among the public was still limited, especially among less-educated groups.^45^^,^^46^ Limited awareness and lack of knowledge about HBV infection and HBV-related diseases is one of the barriers to timely diagnosis and treatment.^39^ Moreover, utilization of antiviral therapy is limited by cost and availability, particularly when patients are not covered by health insurance.^47^ The inequalities in socioeconomic levels and disparities in the proportion of health insurance payments of different regions affect the availability and compliance of antiviral therapy in China and other developing countries. The Chinese government should further raise the awareness and knowledge of HBV testing and treatment among the public and improve reimbursement policies or set up a special government fund to improve affordability of care and hence patient compliance.

In conclusion, China has made good progress on reducing HBV incidence in the past three decades. However, the country still faces challenges to achieve its target of 65% reduction on mortality by 2030. To eliminate the gap in mortality, we suggest that priority should be given to achieving the service coverage targets of diagnosis and treatment. The Chinese government needs to (i) improve the diagnostic and treatment abilities of medical institutions and health workers; (ii) reduce the health-care financial burden for patients; and (iii) integrate existing national programmes and resources to establish a system that covers prevention, screening, diagnosis and treatment of HBV infection across the life cycle. In this way, progress can be made towards the target of eliminating hepatitis B by 2030 in China.
